# Improved Motion Classification With an Integrated Multimodal Exoskeleton Interface

**DOI:** 10.3389/fnbot.2021.693110

**Published:** 2021-10-25

**Authors:** Kevin Langlois, Joost Geeroms, Gabriel Van De Velde, Carlos Rodriguez-Guerrero, Tom Verstraten, Bram Vanderborght, Dirk Lefeber

**Affiliations:** ^1^Robotics & Multibody Mechanics Research Group, MECH Department, Vrije Universiteit Brussel, Brussel, Belgium; ^2^IMEC, Leuven, Belgium; ^3^Flanders Make, Lommel, Belgium

**Keywords:** human-machine interface, classification, exoskeletons, machine learning, intention recognition, electromyogram, wearable sensor

## Abstract

Human motion intention detection is an essential part of the control of upper-body exoskeletons. While surface electromyography (sEMG)-based systems may be able to provide anticipatory control, they typically require exact placement of the electrodes on the muscle bodies which limits the practical use and donning of the technology. In this study, we propose a novel physical interface for exoskeletons with integrated sEMG- and pressure sensors. The sensors are 3D-printed with flexible, conductive materials and allow multi-modal information to be obtained during operation. A K-Nearest Neighbours classifier is implemented in an off-line manner to detect reaching movements and lifting tasks that represent daily activities of industrial workers. The performance of the classifier is validated through repeated experiments and compared to a unimodal EMG-based classifier. The results indicate that excellent prediction performance can be obtained, even with a minimal amount of sEMG electrodes and without specific placement of the electrode.

## 1. Introduction

Upper body exoskeletons for industrial workers have been developed at an increasing pace over the past years and have shown promising results in a controlled lab environment, yet more nuanced for in-field experiments (De Looze et al., 2016; De Bock et al., 2020). Passive devices such as the Paexo (Ottobock, Duderstadt, Germany) (Maurice et al., 2019) or the Mate (Comau, Grugliasco, Italy) (Pacifico et al., 2020) provide assistance by storing energy in elastic elements. This energy is harvested through human motion and can be used to support a specific motion or posture such as overhead working. Due to the passive nature of the device, the assistance level cannot be dynamically controlled limiting the versatility of these devices. Active devices, on the other hand, comprise actuators (such as electric motors or other types, Gopura et al., 2016) which have the potential to deliver different assistive profiles for different tasks (Gull et al., 2020). Providing the right assistance is quite challenging, since the range of motions and tasks humans perform with the upper body is virtually infinite.

To solve this problem, researchers are developing intention recognition and task classification strategies to enable natural control. These recognition algorithms are most often based on uni-modal sensing strategies, most often comprised of myoelectric signals (Kiguchi and Hayashi, 2012; Novak and Riener, 2015; Bi et al., 2019).

Classically these signals are acquired from wet Ag/AgCl electrodes. This method requires to first locate the ideal position for the sensor using body landmarks, then clean and shave the skin at this location and apply a gel to the muscle body from which the signal is collected. For high signal quality, this process requires expertise and a relatively long setup time. When used in combination with an exoskeleton, the optimal locations for the sensors are often obstructed by the interface. In addition, the comfort of the user could be compromised because many of the devices associated with this acquisition technique are bulky and several cables are attached to the arms of the users. These issues reduce the practical applicability of the method for an upper body exoskeleton for industrial workers. Recently, dry electrodes were developed that do not need gel, reduce setup time and allow for more portability. They however come at the cost of a lower signal to noise ratio (Hakonen et al., 2015), which will inevitably reduce classification accuracies. A promising method to overcome the shortcomings of EMG sensors is to combine information from different sensor modalities (Novak and Riener, 2015). In that context, a popular control method for exoskeletons is to combine the information from EMG- and mechanical sensors such as inertial measurement units (accelerometer and gyroscope) or force- and torque sensors.

A sensor that has yet to be combined with EMG are pressure sensors located at the physical interface of exoskeletons. The integration of pressure sensors in physical interfaces has been investigated in the robotics community (De Rossi et al., 2011; Tamez-Duque et al., 2015; Wilcox et al., 2016; Langlois et al., 2021) and have shown their relevance for the unimodal detection of the user motion intention (Lenzi et al., 2011). Additionally, pressure sensors can ensure a more safe and comfortable operation of such devices (He et al., 2017).

Pressure and EMG sensors were combined in wearable bands developed for the detection of hand and wrist motions (Connan et al., 2016; Jiang et al., 2020). The band comprises sEMG electrodes and force sensitive resistors that measure volume changes induced by muscular activity. Fusion of both modalities showed promising results for the performance of gesture recognition. In the domain of upper body exoskeletons, several muscle groups are usually targeted to achieve good recognition (Trigili et al., 2019). However, in the context of industrial workers, the application of electrodes on multiple muscles can be problematic since clothing is usually covering most of the muscles. Wearing t-shirts is still conceivable for certain applications such as logistics. In that regard, only the biceps and triceps muscles are potential sources of EMG information.

In this manuscript we propose a novel integrated, multimodal interface comprising EMG electrodes that measure activity of the biceps brachii, and pressure sensors that monitor the interaction between the user and the exoskeleton. The novelty of this research lies in the combination of EMG- and pressure-signals that this interface can obtain. Moreover, the EMG electrodes as well as the pressure sensors are all 3D printed. This allows to develop the interface for other body regions as well, or to customize the design to a specific user (Langlois et al., 2018).

Experiments on human subjects are carried out to explore the potential of the interface to classify lifting and reaching tasks. The analysis is performed in a test bench consisting of a torque controlled cobot. A classifier based on K-Nearest Neighbours (KNN) algorithm is trained to recognize lifting and reaching tasks in an off-line manner.

## 2. Methods

### 2.1. Sensorized Interface

The physical interface is an upper-arm orthosis with integrated pressure sensors and EMG-electrodes. The objective of such an interface is to ensure the correct placement of the exoskeleton relative to the body, achieve effective force transmission, and most of all, support safe and comfortable interactions. Interface dynamics are known to play a crucial role in the ability of exoskeletons to provide assistance and comfort (Cherry et al., 2016; Langlois et al., 2020).

**Figure 1:**
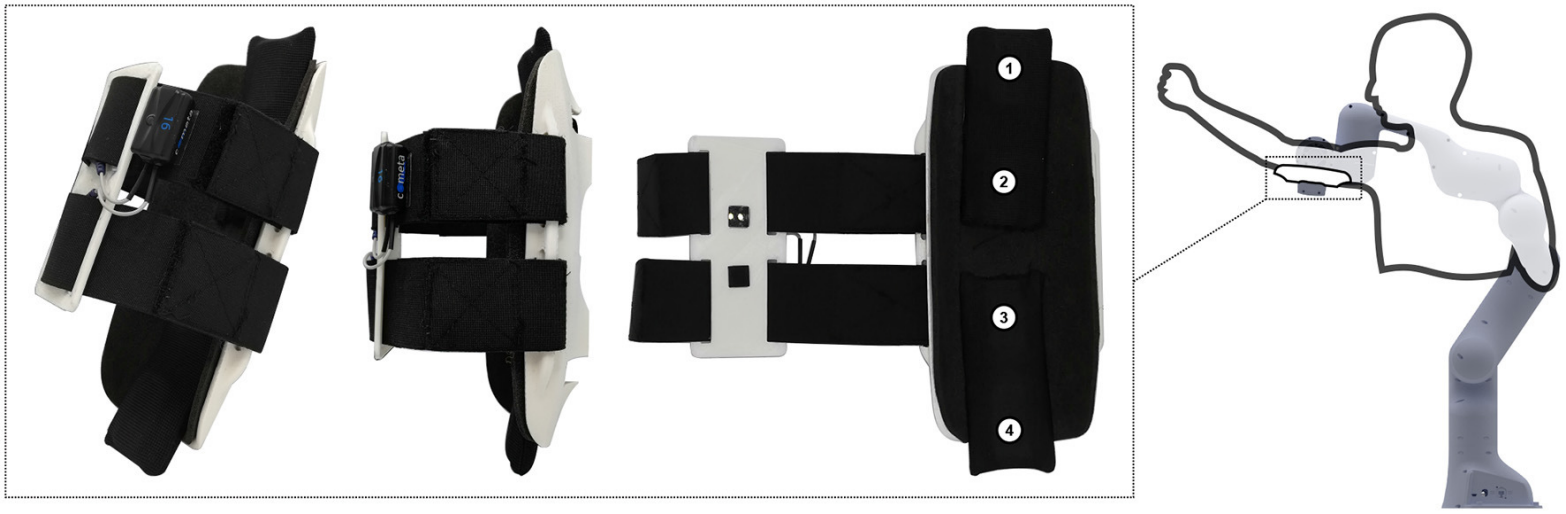
The setup of the experiment consists of a sensorized interface and a torque controlled cobot. The four pressure sensors located on the inside of the interface are shown (number 1–4). The EMG electrodes are the two black squares on the inside of the straps. The cobot simulates an upper body passive exoskeleton using a joint impedance controller.

Four flexible polymer capacitive pressure sensors are integrated along the centre line of the orthosis, shown in Figure 1. The pressure sensors allow pressure measurements with a relative accuracy of approx. 10% at a rate of 10 Hz. At the beginning of every trial the sensors are calibrated relative to the force sensing of the cobot. The design of the pressure sensors and the calibration process are described in detail in Langlois et al. (2021).

In the developed interface, the muscular activity of a single muscle group, the biceps brachii, is monitored. The biceps brachii was chosen since the application for which this interface is designed is the assistance of upper body reaching and lifting during industrial workers' tasks. In that context, the muscles around the shoulder are more challenging to access. Monitoring the triceps muscles as these interact with the interface was out of the scope of this paper. Though co-located force and EMG sensors have been developed (Jiang et al., 2020).

The interface's flexible straps conceal an individual electrode pair, consisting of a printed polylactic acid, conductive filament, similar to Wolterink et al. (2020). The electrodes are pressed against the skin by the elastic straps to ensure skin contact. The same straps are also providing the attachment between the human and the robot. These are tightened the same way a non-sensorized strap would.

Specific electrode placement is not required/performed when donning as the straps ensure a similar pose across users. This approach would be beneficial in a commercial application for industrial workers, where workers do not need expertise or support for donning the exoskeleton. Although resulting in a wider variability of the EMG signal, we believe the pressure sensor data can potentially compensate for this effect. However, once the interface is worn, one should avoid the slippage of the electrodes since this will create noise.

### 2.2. Experimental Setup

The experimental setup comprises the sensorized interface and a torque-controlled cobot (Panda, Franka Emika, Munich, Germany). The cobot is programmed to simulate a passive upper body exoskeleton by means of a joint impedance controller. The impedance controller is set such that an assistive force is exerted onto the interface, effectively pushing the user's arm to an upward position. This means the subject exerts a force to pull the arms down. Similarly to an actual passive exoskeleton, the goal is to assist humans by reducing the efforts delivered at the shoulder level when executing lifting tasks.

**Figure 2:**
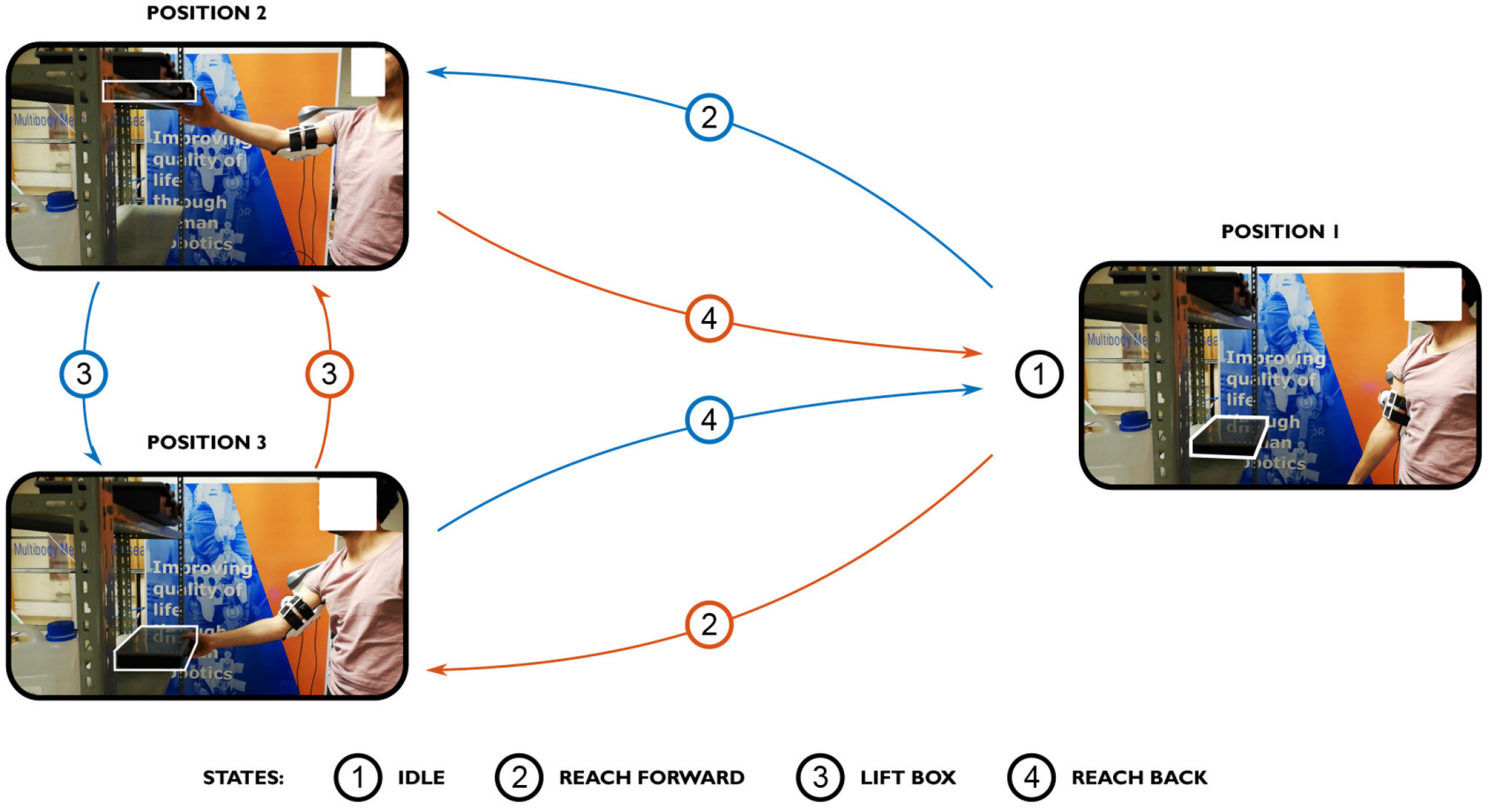
The subject starts in a relaxed position with the arm along the trunk (IDLE). Then, the subject reaches forward (RF) to grab the box, moves the box toward the other shelf (LB), releases the box and reaches backwards (RB). Note that there are two possible initial positions of the box, and thus two paths that can be initially chosen from, as indicated in blue and red.

The experiment consists of two shelves and a box with a mass of 2.2 kg. The user straps him/herself into the interface and performs the task depicted in Figure 2. The task consists of four parts: first, the subject starts the exercise by relaxing the arm along the trunk (IDLE). Then, the subject reaches forward to grab the box on the shelf (RF), grabs and lifts the box (LB) and places it onto the other shelf. The initial position of the box is randomized (top or bottom shelf). After placing the box, the participant reaches back (RB) to idle position and repeats the task. IDLE, RF, LB, and RB are the labels of the exercise. This exercise is repeated during 1 min, at self-selected speed. Each participant repeats the exercise seven times.

During this exercise the muscular activity of the biceps, the pressure acting on the inside of the interface and the position and forces at the end-effector are recorded. The details of the processing methods are explained further below in section 2.3.

### 2.3. Acquisition System

EMG signals from the biceps brachii are sampled through a Cometa Mini Wave Infinity system (Cometa Srl, Bareggio, Italy) at a frequency of 2 kHz. The signals are band pass-filtered (15–400 Hz) before segmentation. A sliding window segmentation is implemented with a window of 600 samples (300 ms) and an overlap of 150 samples.

The pressure data are captured at a 10 Hz rate and the signals are filtered through a second-order Butterworth filter with a cutoff frequency of 2 Hz. The initial pre-compression/loading of the interface is measured and accounted for: At the start of the experiment, the user keeps his/her arm at the equilibrium position of the cobot, i.e., where forces are small, and this for 20 s. The mean pressure over that time window is subtracted from all subsequent measurements.

The force and position measurements at the end-effector of the cobot are captured at a 1 kHz rate and are filtered through a first-order Butterworth filter at a 2 Hz cutoff frequency. The cobot can measure external forces with a resolution of 0.1 N.

Data analysis is performed in the Matlab environment (MathWorks, Massachusetts, United States).

### 2.4. Subjects

A total of 4 healthy subjects participated in the experiment, and they all provided written informed consent. The procedures were approved by the Institutional Review Board at The UZ Brussel, Vrije Universiteit Brussel and complied with the principles of the declaration of Helsinki.

### 2.5. Classification Features

Regarding EMG signals, the 10 following features were selected for further investigation: Root Mean Square (RMS), Wavelength (WL), Enhanced Mean Absolute Value (EMAV), Average Amplitude Change (AAC), Variance (VAR), Simple Square Integral (SSI), Mean Absolute Value (MAV), Integrated EMG (IEMG), Slope Sign Change (SSC), and Zero Crossing (ZC). In terms of pressure, seven features are proposed for further analysis: Pressure values at sensor element 1-4 (P1-P4), Total Pressure (P TOT), Differential Pressure (dP), and Center Of Pressure (COP). The external force (F) acting normal to the interface and the position (X,Z) of the end-effector are also evaluated for further analysis. This constitutes a total of 20 features.

**Figure 3:**
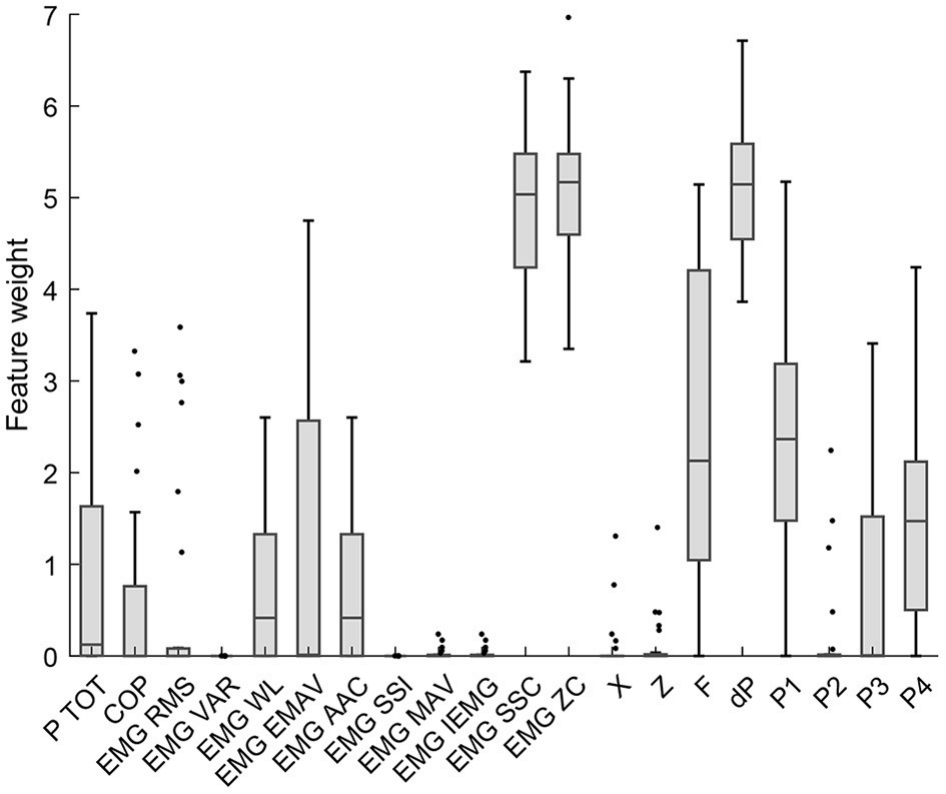
Neighborhood component analysis allows to select features to achieve maximal prediction accuracy. A lower score means a more redundant signal that does not contribute to a higher classification accuracy.

Neighbourhood component analysis (NCA) is performed to reduce the features that are passed on to the classifier. NCA is a non-parametric method for selecting features with the goal of maximizing prediction accuracy of classification algorithms (Yang et al., 2012). The output of the algorithm is a feature weight vector that maximizes the classification accuracy. This algorithm is implemented in Matlab using the *fscnca* function. The results of the analysis are shown in Figure 3. Features with a low score are not considered further. These are the RMS, VAR, SSI, MAV, and IEMG features of the EMG signals, and the position signals of the end-effector.

### 2.6. Classification Protocol

Each subject except one carried out seven trials (due to technicalities, one was discarded for subject four). Each trial is composed of several cycles (ranging from five to eight), as shown in Table 1. All the data was manually labeled based on the speed of the end-effector and the video footage of the experiment. To train the classifier, every trial of each subject is divided into three subsets: a training-, validation- and test set. First, a training set and a test set are divided by leave-one-cycle-out partitioning. This means the classifier is trained (and validated) on all but one cycle. The classifier is then tested on the left-out cycle. This process is repeated for each cycle of each trial, and the average accuracy for each subject is reported in the section 3. The validation set is partitioned based on a hold-out fraction of 25% of the training set.

**Table 1:** Average data across all trials.

**Subject**	**1**	**2**	**3**	**4**
IDLE duration [min max] [s]	2.48 [1.60–3.59]	1.25 [0.63–2.14]	0.97 [0.44–1.88]	0.88 [0.29–1.51]
RF duration [min max] [s]	1.70 [1.07–2.28]	2.36 [1.65–3.79]	1.71 [1.17–2.33]	1.77 [0.92–2.53]
LU duration [min max] [s]	3.10 [2.09–3.89]	4.34 [3.55–5.05]	2.76 [2.28–3.40]	3.30 [2.33–4.37]
RB duration [min max] [s]	2.11 [1.51–3.16]	2.48 [1.79–3.45]	1.96 [2.28–1.60]	2.13 [1.46–2.92]
Cycles [min max]	5.14 [5–6]	4.57 [4–5]	7 [7–7]	6.33 [5–8]
Peak force [N]	8.9	26.75	11.95	17.39
Peak pressure [kPa]	8.47	16.2	6.57	8.12
Peak velocity [Vx,Vz] [m/s]	[0.055;0.117]	[0.194;0.119]	[0.215;0.147]	[0.219;0.133]
ROM [range X; range Z] [m]	[0.111;0.279]	[0.232;0.305]	[0.223;0.291]	[0.199;0.279]

*Large variations across subjects can be observed in terms of how the exercise was performed. Subject 2 performed slower motions with wider range of motion and higher forces, whereas subject 1 performed smaller range of motions with lower forces*.

A K-Nearest Neighbour cosine classifier with 1,001 neighbors is chosen for this task. This parameter gave over-all good results and did not overfit the data. Fewer neighbors will result in higher accuracy for a single dataset but entail a less flexible classifier.

### 2.7. Classification Performances

Four performance metrics are shown in the section 3.

**Accuracy** is the fraction of predictions that are correct:


(1) $$\begin{array}{r} Accuracy=\frac{TP+TN}{n} \end{array}$$

with TP the number of True Positives, TN the number of True Negatives and n the number of predictions.

**Sensitivity** or true positive rate measures the proportion of positives that are correctly identified:


(2) $$\begin{array}{r} Sensitivity=\frac{TP}{P} \end{array}$$

with P the number of real positive cases in the data.

**Specificity** or true negative rate measures the proportion of negatives that are correctly identified:


(3) $$\begin{array}{r} Specificity=\frac{TN}{N} \end{array}$$

with N the number of real negative cases in the data.

## 3. Results

### 3.1. Motion Data, Pressure, and Force

Since the four participants could perform the task at a self-selected speed, we found widely varying executions, both in terms of speed of execution and forces acting on the body as well as muscular activity. First, in terms of external force acting normal to the interface, we observed varying peak forces across the subjects. For subject one, the lowest peak force is found, with a value of 8.9 N. The highest peak force is found for subject 2, with a value of 26.75 N. The forces acting on the interface depend on the equilibrium position of the robot, which had to be slightly adjusted for each participant, as well as the range of motion (ROM) of the participant. Since the robot acts as an impedance, the further away from equilibrium a subject moves the arm, the higher the force. The smallest range of motion is found for subject 1, with a total range across all trials of 0.111 m along the X-direction (moving the hand forward, parallel to ground) and 0.279 m in the Z-direction (parallel to gravity). The highest range of motion is found for subject 2, with a total range of 0.232 m and 0.305 m, in the X- and Z-direction respectively. In terms of pressure, the peak occurs during the lifting motion of the box. This peak pressure is caused by the assistive force of the robot and the change in volume of the arm. The lowest peak pressure is found for subject 3, with a value of 6.57 kPa. The highest peak pressure is found for subject 2, with a value of 16.2 kPa. At the same time, subject 3 spent the least amount of time lifting up the box (LU), on average. While subject 2 spent, on average, the most amount of time lifting up the box. Subject 2 also performed the least amount of cycles per trial, with an average number of cycles of 4.57.

**Figure 4:**
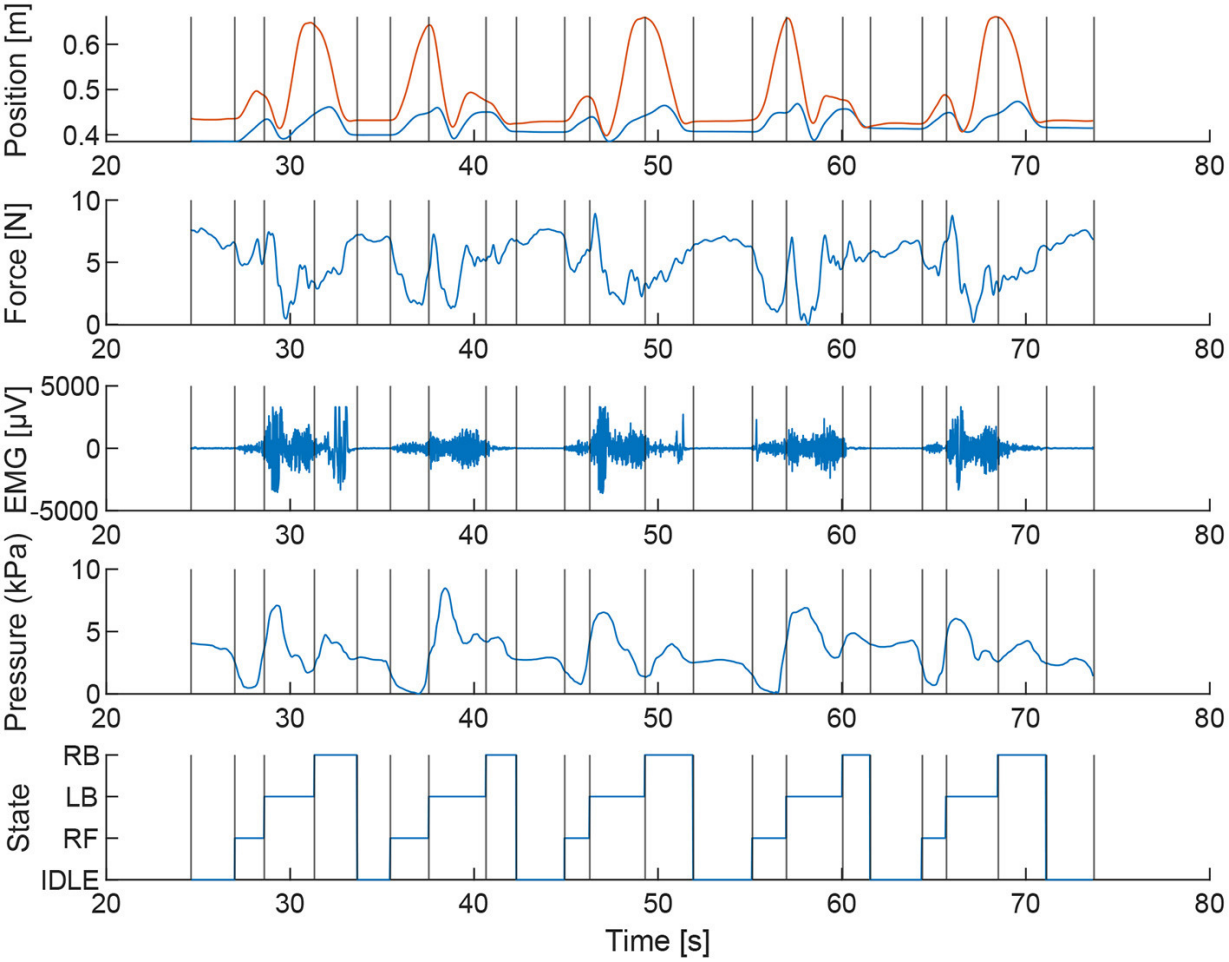
During the task the motion and force of the end-effector, the pressure inside the interface and the muscular activity of the biceps brachii are sampled. The data shown are the results of one of the trials of subject 1.

All the average values across all trials are shown in Table 1. In Figure 4 the raw data outcome of a single trial of subject 1 is shown.

### 3.2. Neighbourhood Component Analysis

The NCA revealed the features that are redundant in the classification of the tasks. The features that were not further used for classification are the RMS, VAR, SSI, MAV, and IEMG features of the EMG signals, and the position signals of the end-effector. The P2 feature was left in the feature pool. The two outer sensors, P1 and P4 scored a higher feature weight (median weight of 2.4 and 1.5, respectively) than the inner pressure sensors, P2 and P3 (median weight of 0.0 for both). The Zero Crossing feature and the Slope Sign Change (SSC) are the two highest rated features of the EMG signals, with a respective median weight of 5.2 and 5.0. The differential Pressure (dP) was the second highest feature of all, with a median score of 5.1.

Based on these results, seven classifiers are further analyzed. First, three unimodal classifiers (i.e., single type of sensor) are constructed: the EMG-classifier (comprising WL, EMAV, AAC, SSC and ZC features), the P-classifier (comprising P TOT, COP, dP, P1, P2, P3, P4 features) and the F-classifier (comprising F feature). Then, multimodal classifiers based on all combinations of sensors are constructed: EMG+P, EMG+F, P+F and EMG+P+F.

### 3.3. Classifier Performances

#### 3.3.1. EMG, Pressure, and Force

**Figure 5:**
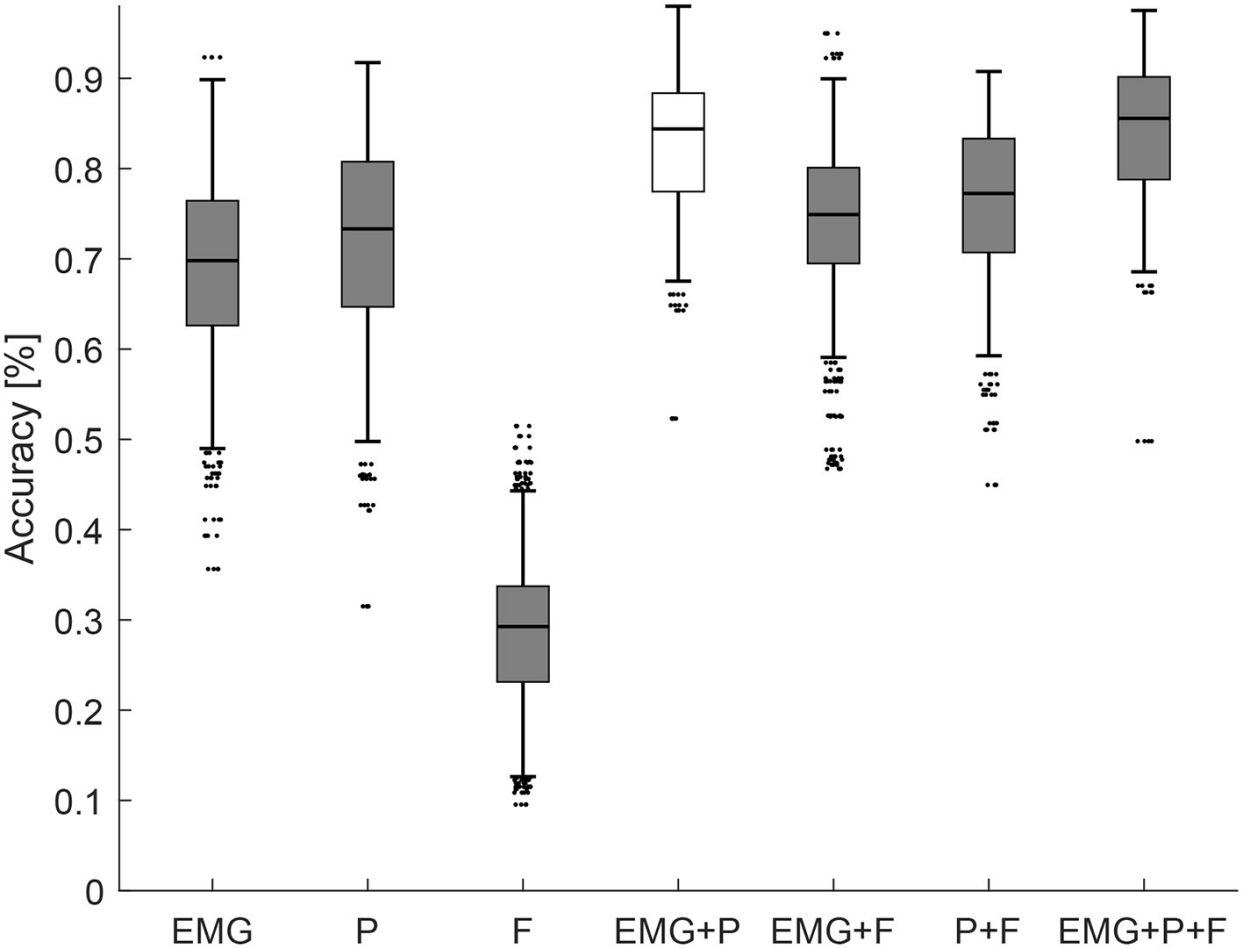
Accuracy of all the classifiers across all subjects and trials trained on different sensory input.

Across all participants and all trials the highest median accuracy is achieved with all the features, as shown in Figure 5, with a value of 85.6 %, followed by the EMG+P classifier, with an accuracy of 84.4 %. The P classifier scored better than the EMG classifier with a value of 73.3 % and 69.8 % respectively. The classifier based on external force only scored the worst result with a value of 29.3 %. The best classifier using only two modalities is the EMG+P classifier which is further analyzed below.

#### 3.3.2. EMG and Pressure

**Figure 6:**
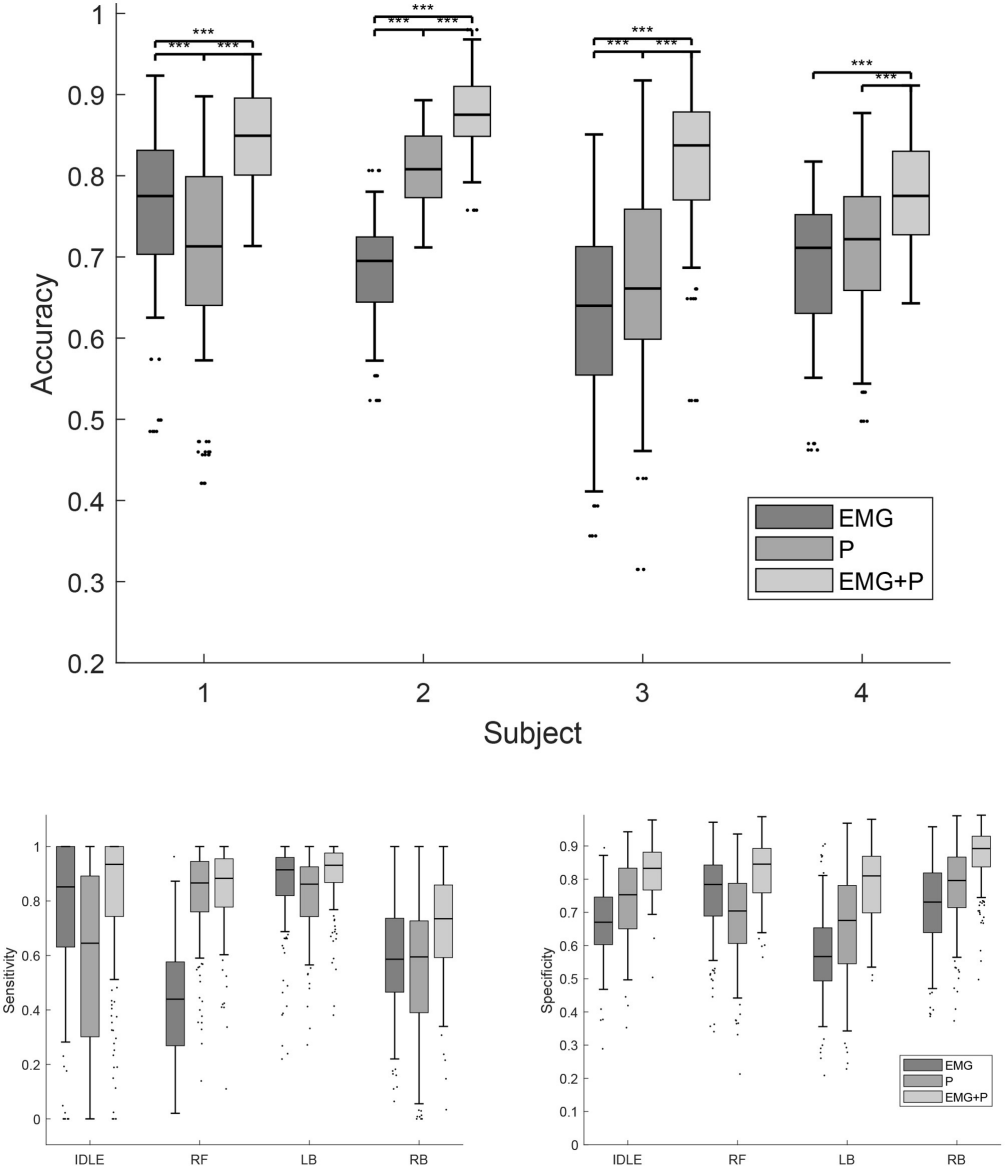
The accuracy, sensitivity, and specificity of the EMG, P, and EMG+P classifiers. The dots indicate outliers, the stars indicate levels of significance.

**Figure 7:**
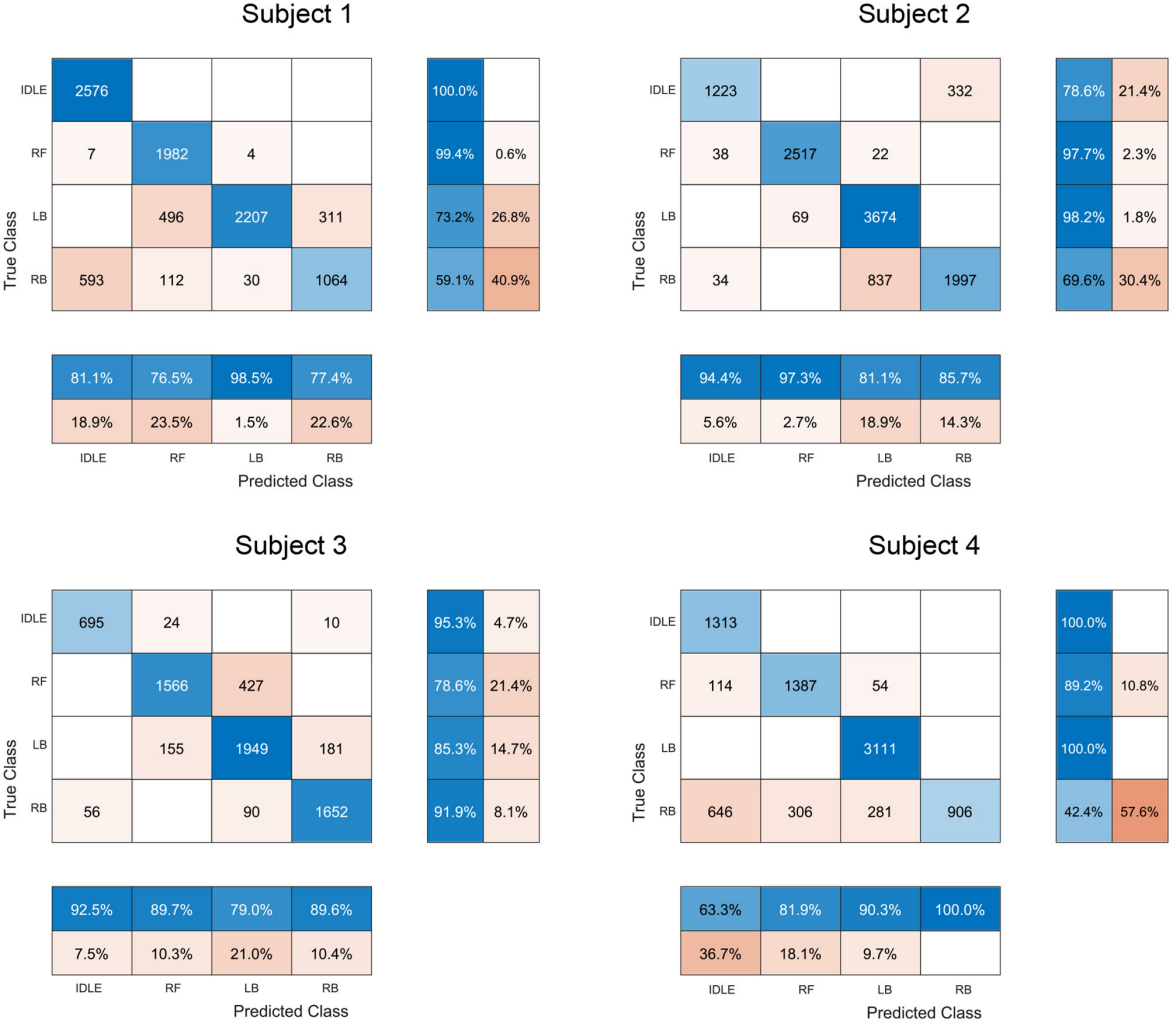
Confusion matrices for the EMG+P classifier for each subject. The row summaries display the percentages of correctly and incorrectly classified observations for each true class. The column summaries display the percentages of correctly and incorrectly classified observations for each predicted class. Diagonal and off-diagonal cells correspond to correctly and incorrectly classified observations, respectively.

A Tukey-Kramer *post-hoc* test showed a statistically significant better result with the multimodal classifier relative to the uni-modal classifiers across all participants and trials (p ≤ 0.0001). For subject 1 a statistically significant better result was achieved with the EMG classifier, relative to the P classifier (*p* ≤ 0.0001). For subject 2 (*p* ≤ 0.0001) and subject 3 (*p* ≤ 0.001) the opposite is true. For subject 4 the P classifier was not statistically better than the EMG classifier (*p* ≤ 0.052). These results are reported in Figure 6. In Figure 7, the confusion matrices of the EMG+P classifiers are shown for the four subjects. The confusion matrices shown are the results from trial 6, 13, 20, and 23 which represent closely the median accuracies reported in Figure 6.

## 4. Discussion

The main outcome of the analysis is that pressure is a relevant sensory input that can be combined with EMG sensors to recognize lifting and reaching tasks. To the author's knowledge it is the first time this combination of sensors is proposed for the recognition of upper body tasks while wearing an upper body assistive device. The peak classification accuracy found across all subjects and trials is 96.8 %. An important limitation of this outcome is that the classifiers trained in this study are subject specific. For a practical implementation of such devices a non-subjective specific classifier is preferred. Trigili et al. (2019) demonstrated the implementation of such a recognition algorithm through EMG signals alone. As much as seven muscle groups of the upper body were targeted to achieve good classification performances. While it would not be practical to integrate that many electrodes in a commercial device, perhaps a multimodal strategy such as the one described in this manuscript can help reduce the number of necessary electrodes.

Another limitation, are the performances reported in this study, which are achieved through an off-line classification process. It is known from literature that on-line classifiers do not perform as well (Novak and Riener, 2015). For on-line classifiers the processing time, as well as the feature extraction windows become more critical, since dynamic tasks require fast decision making. In that aspect a trade-off between classification accuracy and timing is inevitable. Different lengths of classification windows are known to affect classification performance (Smith et al., 2010). Potentially, in the case of on-line classification a KNN-based classifier might become a bottleneck, since the classifier relies on the calculation of angles between all neighbors for each new feature sample. Consequently, the storage requirements and the computational time proportionally increase with the size of the training set (De Leonardis et al., 2018). A variety of other classifiers such as linear discriminant analysis (LDA), support vector machines (SVM), decision trees (DT), or artificial neural networks (ANN) have been used in the literature (Novak and Riener, 2015; Bi et al., 2019) and are not showing the same disadvantages (De Leonardis et al., 2018).

Since the sample size was small, only subject specific classifiers were developed. For a general classifier, a large sample size will be required, and a different fusion algorithm might be necessary to cope with the variability. More specifically, it is known that the variability of EMG signals can be significant across time due to artefacts and crosstalk (Bi et al., 2019). On the other hand, we found that pressure measurements are generally more stable signals across a particular pattern, which is in line with previous research on the topic (Connan et al., 2016; Langlois et al., 2021). This leads us to expect the pressure sensing would improve a more general motion classification across a large sample size as well. Interestingly, in our experiments the classifier based solely on external force did not perform as well as expected. Most probably, adding the shear component of force (instead of only the normal component) would improve the results. Also adding a derivative or integral component of force to the features might improve the performance, albeit with the necessary filtering.

The same observation holds true for the position information of the end-effector. The neighbourhood component analysis determined the position data to be redundant with regard to the other modalities. Even though, measuring limb position was shown to increase classification accuracy (Fougner et al., 2011), since it resolves the position effect (Radmand et al., 2014). Potentially, the pressure sensors carry information about the position of the limb since the robot is programmed to exert an assistive force by means of a joint impedance controller. Additionally, adding a derivative and second derivative term of the position might result in a different outcome.

Several potential further developments could lead to improved performance of the presented design. Firstly, a similar design to the one presented in Jiang et al. (2020), wherein a co-located force sensor and EMG sensor is implemented would allow the triceps muscles to be monitored as well. While contact might not always be ensured in an exoskeleton, the system can be trained to recognize electrode displacements and mitigate losses in classification accuracy (Hargrove et al., 2008). In that regard, assessing how pressure readings can further improve detection of electrode shift can be interesting. Secondly, to limit complexity, a single electrode pair was printed in the interface. In the future an array of electrodes could be integrated which could compensate for the problem of lower signal to noise ratio. Thirdly, the classification in this experiment was achieved using a KNN algorithm. This type of algorithm was chosen since it is considered a simple and efficient method that yields competitive results compared to state-of-the-art classification methods (Yang et al., 2012; De Leonardis et al., 2018). Previous research has shown that the effects of algorithm type on accuracy is generally small for single time invariant classifiers, and the choice of specific features seems to be more important (Novak and Riener, 2015). However, other types of classification methods such as adaptive or parallel classifiers should be considered in the future, since superior classification accuracy for myoelectric control was shown, albeit at the expense of added complexity (Novak and Riener, 2015).

## 5. Conclusion

In this paper an integrated multimodal interface for upper-body exoskeletons is presented. The analysis shows the relevance of integrating pressure sensors and EMG sensors into interfaces with the aim of improving classification of upper body lifting and reaching tasks. The performed neighbourhood component analysis revealed that the WL, EMAV, AAC, SSC and ZC features of the EMG signal, the dP, P TOT, COP, P1-P4 features of the pressure signals and the external force provided the most information toward optimal classification. In the future, researchers in the field should look into the possibility of developing smarter interfaces, integrating different sensors to achieve better recognition algorithms.

## Data Availability Statement

The data presented in this study are available on request from the corresponding author.

## Ethics Statement

The studies involving human participants were reviewed and approved by UZ Brussel, Vrije Universiteit Brussel. The patients/participants provided their written informed consent to participate in this study.

## Author Contributions

KL, JG, CR-G, BV, and DL developed the concept. KL developed the technology with inputs from TV, BV, and DL. KL, JG, and GV conceived and designed the experiments. TV, JG, GV, CR-G, and BV contributed to the design and layout of the article, tables, additions to the bibliography, and extensive revisions. CR-G, BV, and DL helped to obtain the funding for the project that financed this research. All authors contributed to the article and approved the submitted version.

## Funding

The work presented in this paper was supported by the Research Foundation - Flanders (FWO) under grant no. S000118N SBO Exo4Work project. TV was a postdoctoral fellow of the Research Foundation Flanders (FWO).

## Conflict of Interest

The authors declare that the research was conducted in the absence of any commercial or financial relationships that could be construed as a potential conflict of interest.

## Publisher's Note

All claims expressed in this article are solely those of the authors and do not necessarily represent those of their affiliated organizations, or those of the publisher, the editors and the reviewers. Any product that may be evaluated in this article, or claim that may be made by its manufacturer, is not guaranteed or endorsed by the publisher.
